# Molecular Cloning and Characterization of Juvenile Hormone Acid Methyltransferase in the Honey Bee, *Apis mellifera*, and Its Differential Expression during Caste Differentiation

**DOI:** 10.1371/journal.pone.0068544

**Published:** 2013-07-09

**Authors:** Wenfeng Li, Zachary Y. Huang, Fang Liu, Zhiguo Li, Limin Yan, Shaowu Zhang, Shenglu Chen, Boxiong Zhong, Songkun Su

**Affiliations:** 1 College of Animal Sciences, Zhejiang University, Hangzhou, China; 2 Department of Entomology, Michigan State University, East Lansing, Michigan, United States of America; 3 ARC Centre of Excellence in Vision Science, Research School of Biology, College of Medicine, Biology and Environment, the Australian National University, Canberra, Australia; 4 College of Bee Science, Fujian Agriculture and Forestry University, Fuzhou, China; U. Kentucky, United States of America

## Abstract

Juvenile hormone acid methyltransferase (JHAMT) is an enzyme involved in one of the final steps of juvenile hormone biosynthesis in insects. It transfers a methyl group from S-adenosyl-L-methionine (SAM) to the carboxyl group of either farnesoic acid (FA) or JH acid (JHA). Several genes coding for JHAMT have been cloned and characterized from insects from different orders, and they have been shown to play critical roles in metamorphosis and reproduction. However, the significance of *JHAMT* in Hymenopteran insects is unknown. We used RACE amplification method to clone *JHAMT* cDNA from the honey bee, *Apis mellifera* (*AmJHAMT*). The full length cDNA of *AmJHAMT* that we cloned is 1253bp long and encodes a 278-aa protein that shares 32-36% identity with known JHAMTs. A SAM-binding motif, conserved in the SAM-dependent methyltransferase (SAM-MT) superfamily, is present in AmJHAMT. Its secondary structure also contains a typical SAM-MT fold. Most of the active sites bound with SAM and substrates (JHA or FA) are conserved in AmJHAMT as in other JHAMT orthologs. Phylogenetic analysis clustered AmJHAMT with the other orthologs from Hymenoptera to form a major clade in the phylogenetic tree. Purified recombinant AmJHAMT protein expressed in *E. coli* was used to produce polyclonal antibodies and to verify the identity of AmJHAMT by immunoblotting and mass spectrometry. Quantitative RT-PCR and immunoblotting analyses revealed that queen larvae contained significantly higher levels of *AmJHAMT* mRNA and protein than worker larvae during the periods of caste development. The temporal profiles of both *AmJHAMT* mRNA and protein in queens and workers showed a similar pattern as the JH biosynthesis. These results suggest that the gene that we cloned codes for a functional JHAMT that catalyzes the final reactions of JH biosynthesis in honey bees. In addition, *AmJHAMT* may play an important role in honey bee caste differentiation.

## Introduction

Juvenile hormones (JHs) are a group of sesquiterpenoids uniquely present in insects. JHs play fundamental roles in many aspects of postembryonic life, including development, metamorphosis, reproduction, as well as division of labor and caste differentiation in social insects [1-5]. Changes in JH titers in insect hemolymph regulate the physiological functions mentioned above and are predominantly controlled by regulating the rate of JH biosynthesis [6]. JHs are synthesized *de novo* in a specialized endocrine gland, the corpus allatum (CA) [1].

There are several JH homologs, such as JH 0, JH I, 4-methyl JHI, JH II, and JH III in insects [1]. However, JH III is the only isoform found in *Apis mellifera* [7,8]. The biosynthetic pathway of JH III in the CA consists of two parts [1]. The early steps follow the classical mevalonate pathway conserved in both vertebrates and invertebrates that proceeds from acetyl-CoA to farnesyl diphosphate [9]. The late steps of JH biosynthesis are unique to insects and crustaceans. First, farnesyl diphosphate is hydrolyzed to farnesol by farnesyl diphosphate pryophosphotase. Then, farnesol is converted to farnesal and farnesoic acid (FA) by two successive oxidations catalyzed by farnesol oxidase and farnesal dehydrogenase, respectively. Finally, FA is converted to active JH III by two catalytic actions, an epoxidation at sites C10 and 11 and a methylation of the carboxyl group, respectively catalyzed by a P450 monooxygenase and juvenile hormone acid methyltransferase (JHAMT) [9].

The enzymes involved in the late steps are highly specific to insects. In recent years, molecular cloning techniques have greatly facilitated the characterization of these enzymes. The first *JHAMT* gene (*BmJHAMT*) was cloned from the CA of the silkworm, *Bombyx mori* [10] and was found to belong to the S-adenosyl-L-methionine-dependent methyltransferase (SAM-MT) superfamily. The recombinant BmJHAMT expressed in *E. coli* transferred the methyl group from S-adenosyl-L-methionine (SAM) to JHA, as well as FA, resulting in methyl esters, JH III or methyl farnesoate (MF) [10]. There was a strong correlation between the expression levels of *BmJHAMT* and the rates of JH biosynthesis [10]. Transcriptional suppression of *BmJHAMT* was found to be critical for the initiation of metamorphosis [10,11]. Several orthologs of *JHAMT* have been subsequently cloned and characterized in other insects. These orthologs were also predominantly expressed in CA and displayed catalytic properties similar to *BmJHAMT* [12-16]. All studies revealed that *JHAMT* expression levels were highly correlated to the rates of JH biosynthesis, suggesting that *JHAMT* has an important role in regulating JH synthesis. Direct evidence for *JHAMT* function *in vivo* has also increased over time. Overexpression of *JHAMT* in the model Dipteran *Drosophila melanogaster* dramatically prolonged pupal development and resulted in pharate adult lethality and rotation defects in male genitalia [15]. Both of these effects were also observed after the topical application of JH or JH mimic on the wandering 3^rd^ instar wild-type larvae [15]. In addition, RNA interference-mediated knockdown of *JHAMT* in the red flour beetle *Tribolium* *castaneum* caused precocious metamorphosis, which could be rescued by JH or JH mimic treatment [14]. Another study conducted RNA interference of *JHAMT* in the desert locust *Schistocerca gregaria*. Suppression of *JHAMT* transcription levels in this species significantly reduced JH release and resulted in smaller basal oocytes, indicating that *JHAMT* regulates the reproduction of female desert locusts [12].

The biological roles of *JHAMT* orthologs have been studied in several insects from different orders. However, the significance of *JHAMT* in the large insect order Hymenoptera still remains unknown. In this study, we present the molecular cloning and characterization of *Apis mellifera JHAMT* gene (*AmJHAMT*). The recombinant protein of *AmJHAMT* was expressed in *E. coli* and verified by immunoblotting and mass spectrometry. The mRNA and protein levels of *AmJHAMT* were measured during several developmental stages of worker and queen larvae bees.

## Materials and Methods

### Bees

All honey bee larvae and pupae were taken from the *Apis melliferaligustica* colonies (Zhenongda No. 1) maintained in the experimental apiary of Zhejiang University, China (30.272° N, 120.191° E). To obtain precisely-aged larvae, a queen was caged on an empty comb for 6 hours for egg laying. After 72 hrs, the newly-hatched larvae (10-16 hrs old) were transferred into artificial queen cups for queen rearing according to standard apicultural protocol [17]. Worker larvae were collected directly from worker cells. Larvae and pupae of both castes were staged based on previously established criteria [18]. We collected both queen and worker bee samples during the second (L2), third (L3), fourth (L4), fifth (L5) instar larvae stages and the white-eyed pupae stage (Pw). L5 was further divided into the feeding stage (L5F), spinning stage (L5S), and prepupal stage (L5PP). All bee samples were used in the following qPCR experiments and immunoblotting analysis of protein expression. The L5F queen larvae were also used for gene cloning. Samples were instantly frozen in liquid nitrogen and stored at -80°C until use.

### RNA extraction

Frozen bee samples were pulverized in liquid nitrogen with a mortar and pestle. Total RNA was isolated from the powdered tissue using TRIzol reagent (Invitrogen) according to the manufacturer’s protocol. For RNA extraction of L2, L3, and L4 queen or worker larvae, 24, 12, and 6 larvae were pooled respectively for a biological replicate. For all later stages, an individual larva or pupa was used. Three biological replicates were prepared for each developmental stage and caste in the qPCR analysis. RNA sample concentration and purity was measured using a Nanodrop 2000 spectrophotometer (Thermo Fisher Scientific Inc.).

### Cloning full-Length cDNA of *AmJHAMT*

The 5’ and 3’ ends of *AmJHAMT* cDNA were obtained using a SMARTer RACE cDNA amplification kit (Clontech). Total RNA extracted from L5F queens served as a template for the first-strand cDNA synthesis. Primers were designed based on the predicted gene record (GenBank accession: XM_001119986) derived from the *Apis mellifera* genome sequence and were designed to produce DNA fragments with overlapping regions. In addition to the primers supplied in the Clontech kit, primer 5’-GGAAGTTTTGGCACCAGTGAAGGCA-3’ was used for the 5’ RACE and primer 5’-GATACAATACCGTGACGCAGCCGAC-3’ was used for the 3’ RACE. All PCR reactions were performed with an Advantage 2 PCR kit (Clontech) and PCR conditions were as follows: 5 cycles of 94°C for 30 s, 72°C for 3 min; 5 cycles of 94°C for 30 s, 70°C for 30 s, 72°C for 3 min, followed by 27 cycles of 94°C for 30 s, 68°C for 30 s and 72°C for 3 min. Following amplification, the PCR products were gel-extracted and cloned into a pMD19-T vector (Takara Bio, Dalian, China). Several clones for both cDNA ends were selected and sequenced. Finally, the sequences obtained were combined to generate the full length cDNA of *AmJHAMT* using DNAStar software (DNAStar, Inc., WI). To verify the entire sequence, a full-length cDNA with a complete open reading frame (ORF) was subcloned into a pMD19-T vector (Takara Bio, Dalian, China) and sequenced.

### Sequence analysis and phylogenetic tree construction

The protein sequence of *AmJHAMT* was predicted with DNAStar software and its physicochemical properties were predicted by ProtParam [19]. Moreover, the presence and location of signal peptide cleavage sites were detected by SignalP 4.0 [20] and the conserved domain of AmJHAMT was analyzed with the Batch CD-Search tool [21]. Related insect JHAMT sequences were identified by BLAST searches to GenBank [22]. We aligned the amino acid sequences of putative JHAMTs with ClustalX 2.0 [23] and multiple alignments were manually edited in GeneDoc (Free Software Foundation, Inc, Boston, MA). The secondary structure of AmJHAMT was predicted using PredicProtein [24], PRIPRED [25] and Jpred 3 [26]. Consistent structural predictions from these different methods were considered to be strong evidence for secondary structure elements.

A neighbor-joining tree of 24 selected JHAMTs was constructed with Mega 4.0 software [27]. This phylogenetic tree was tested with 1000 bootstrap replicates in Mega 4.0. A Poisson correction was selected as the substitution model and a pairwise deletion method was used for gaps/missing data.

### Preparation of recombinant AmJHAMT

The coding region of *AmJHAMT* was PCR amplified from the cDNA template with primers, 5’-AAAC A T A T GTTCTTGGTCGAGGAATACGTG-3’ and 5’-AAC T C G A GGTTACGACGAATGAAACATTTGTTG-3’. Underlined sequences show the recognition sites of the restriction enzymes *Nde*I and *Xho*I. The PCR product was then subcloned into a pMD19-T vector and sequenced to screen for any PCR amplification errors. The inserted pMD19-T vector and empty pET-28a(+) vector (Novagen) were both digested with *Nde*I and *Xho*I to produce fragments containing the full *AmJHAMT* ORF and a linearized expression vector. These two parts were ligated together, yielding a pET-28a(+)/*AmJHAMT*. The obtained construct was used to transform *E. coli* strain BL21 (DE3). The expression of recombinant AmJHAMT was performed as previously described [10] and the cell pellets were harvested by centrifugation and stored at -20°C. We extracted total protein from the thawed bacterial cell pellets by using a HisTALON buffer set (Clontech). The recombinant His-tagged AmJHAMT protein was purified from extractions with a HisTALON gravity column purification kit (Clontech), following the manufacturer’s instructions. Glycerol was added to the protein solution to obtain a final concentration of 25% and the sample was stored at -80°C until use.

### One-dimensional SDS polyacrylamide gel electrophoresis (SDS-PAGE)

SDS-PAGE was performed in 15% polyacrylamide gel containing 0.1% SDS [28]. The samples were boiled in 1×SDS gel-loading buffer (50 mM Tris·Cl, pH 6.8, 2% SDS, 10% glycerol, 0.1% bromophenol blue, and 100mM DTT) for 5 min at 95°C, centrifuged at 12000×g for 5 min, then subjected to electrophoresis (80 V for stacking gel and 120 V for separating gel). A prestained protein ladder (Fermentas) was used. Gels were stained with Coomassie Brilliant Blue followed by de-coloration in a mixture of 45% distilled water, 45% acetic acid, and 10% methanol.

### Generation of polyclonal antibody and western blotting

The purified recombinant AmJHAMT was used to immunize female rabbits *Chinchilla* *bastard* . The obtained polyclonal antiserum was tested for antibody titer and specificity. It was purified on an affinity column with immobilized AmJHAMT protein.

Western blotting analysis was conducted on size-separated proteins electrophoretically transferred onto polyvinylidene fluoride (PVDF) membranes (Immobilon-P; Millipore, Billerica, MA, USA). The blotted membranes were blocked with 5% milk solution (non-fat milk powder dissolved in TBST), then incubated for 2 h with the obtained AmJHAMT antibody diluted 1:32000. After three washes (10 min per wash) with TBST, the membranes were incubated for 1 h with anti-rabbit alkaline phosphatase-conjugated antibody diluted 1:10000 (Sigma, St. Louis, USA). Visualization of immunoreactive proteins was performed with chemiluminescent NBT/BCIP substrate.

### Liquid chromatography-tandem mass spectrometry analysis

We verified the recombinant AmJHAMT by liquid chromatography-tandem mass spectrometry (LC-MS/MS) analysis. First, we ran the purified recombinant protein on a 15% SDS-PAGE gel and stained the gel with coomassie brilliant blue. Then, we excised the protein band and digested it with trypsin. The tryptic peptides were freeze-dried and dissolved in 0.1% formic acid.

LC-MSMS was performed using a LTQ DecaXP plus mass spectrometer (Thermo Finnigan, San Jose, CA, USA) fitted with a C18 reverse phase column (15cm×150µm, CTI, CA, USA). For liquid chromatography, the compositions of the two mobile phases were as follows: 0.1% formic acid in water for A and 0.1% formic acid in aqueous 80% acetonitrile (ACN) for B. The tryptic peptides were separated by gradient elution as follows: mobile phase B was increased from 5–60% (from 0 to 30 min), followed by a rapid increase from 60–100% (from 30 to 35 min). B was then sustained at 100% for 5 min (from 35 to 40 min). Finally, B was quickly decreased from 100–5% (from 40 to 45 min). The LTQ DecaXP plus mass spectrometer was set as one full MS scan followed by three MS^2^ scans. The MS/MS raw data were searched against the *Apis mellifera* protein records in all NCBI databases and the deduced AmJHAMT sequence obtained by RACE amplification using the TurboSEQUEST program in BioWorks 3.2 (Thermo Finnigan, San Jose, CA, USA). The filtering parameters were set as described previously [29].

### Quantitative real time PCR (qPCR)

A two-step qPCR was used to quantify gene expression in this study. cDNA synthesis was performed from 1 µg total RNA using the ReverTra Ace qPCR RT Kit (TOYOBO, Osaka, Japan). Primers for *AmJHAMT* were as follows: 5’-TATGTATCACGACGAGGA-3’ and 5’-GAATGCTTTCTGGAAGTTT-3’, designed with Primer Premier 6.0 (PREMIER Biosoft International, Palo Alto, CA), and the amplicon was 142 bp. The *Apis mellifera Actin* gene (GenBank accession number: XM_623378) was selected as a reference gene and its primers, 5’-TGCCAACACTGTCCTTTCTG-3’ and 5’-AGAATTGACCCACCAATCCA-3’, were adopted from previous studies [30]. The amplicon was 149 bp. To validate the primer pairs, serial 8× dilutions of cDNA sample were utilized to generate relative standard curves and amplification efficiencies (*E*) and correlation coefficients (r) were calculated for each primer pair. This resulted in an *E* value close to 1 (*E* = 10^(-1/slope)^ – ^1^ [31]), as well as *r* ^2^> 0.99 for both primer pairs (Figure S1). All qPCR reactions were performed in triplicate as technical replicates on a Mastercycler® EP RealPlex System (Eppendorf) using SYBR Green detection. Each 20 µl reaction was made up of 10 µl THUNDERBIRD SYBR qPCR Mix (TOYOBO, Osaka, Japan), 0.6 µl forward primer (10 mM), 0.6 µl reverse primer (10 mM), 2 µl 50-fold diluted cDNA solution and 6.8 µl nuclease-free water. PCR conditions were 95°C for 2 min, followed by 40 cycles of 95°C for 15 s, 55°C for 20 s and 72°C for 45 s. Melting curves and negative control reactions (using nuclease-free water as templates) were monitored to assess amplification specificity and DNA contamination. Only a single peak was found in the melting curves of all experimental reactions, while no peaks emerged in negative controls. PCR products were run on a 2% agarose gel to confirm a product of the correct size. Realplex software (Eppendorf) was used to calculate the threshold cycle (C_T_) values, then the mean C_T_ of three technical replicates was used to quantify the relative *AmJHAMT* expression using the comparative C_T_ method [32].

### Profiling the protein expression by immunoblotting

First, all bee samples were freeze-dried for precise tissue-weighing. 0.08g of each sample was pulverized in liquid nitrogen with a mortar and pestle. The prepared tissue powder was lysed with 300µl RIPA lysis buffer (Beyotime, Nantong, China) on ice for 30 min. The lysate was then centrifuged at 4°C, 12,000 × g for 30 min and the supernatant was carefully transferred to a clean tube. We determined the total protein concentration of each sample by using a Nanodrop 2000 spectrophotometer (Thermo Fisher Scientific Inc.) and adjusted all samples to the same protein concentration. The SDS-PAGE and immunoblotting analyses were carried out as described above. The differences were that the AmJHAMT antibody was diluted 1:1000, and β-actin was selected as an internal reference and its antibody (Huabio, Hangzhou, China) was diluted 1:2000. The loading quantity of samples was also adjusted according to the detected β-actin protein levels until they reached the same level.

### Statistics

Student *t*-tests were used to determine the significance of *AmJHAMT* expression differences between queen and worker larvae and pupae at the same developmental stages. Two-tailed probabilities were adopted in the tests. All analyses were performed with PASW Statistics 18 software (SPSS Inc., Chicago, IL, USA).

## Results

### Cloning and characterization of *AmJHAMT* gene

**Figure 1:**
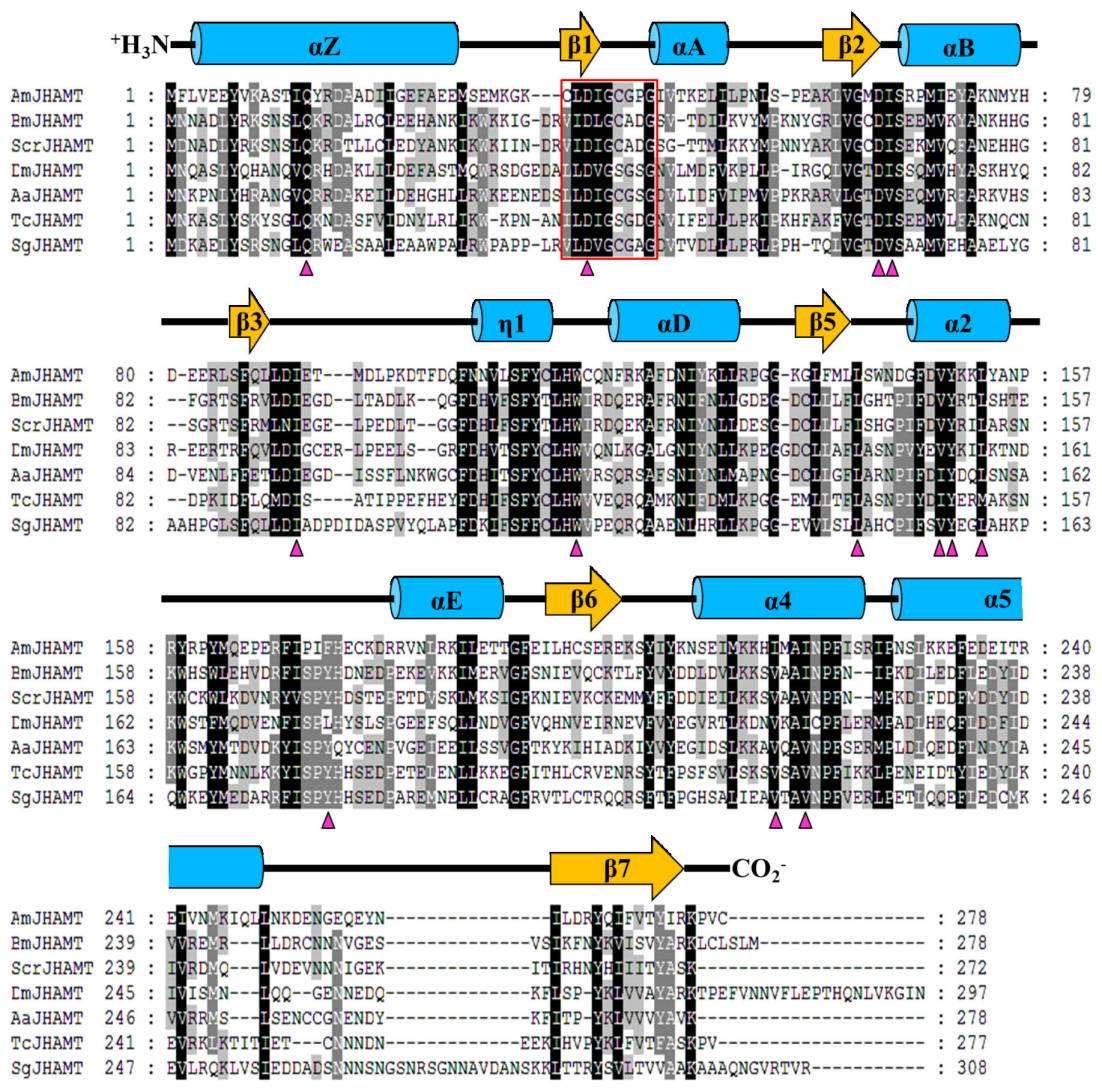
Multiple alignment of seven insect JHAMTs. Species abbreviations included in the sequence labels are as follows: Sg, *Schistocerca gregaria*; Tc, *Tribolium* *castaneum* ; Dm, *Drosophila melanogaster*; Aa, *Aedes aegypti*; Bm, *Bombyx mori*; Scr, *Samia cynthia ricini*; Am, *Apis mellifera*. The conservative properties of amino acid residues are displayed using four mark patterns, the white letters with dark background, white letters with grey background, black letters with grey background and black letters without background, indicating the conservation going from the highest to the lowest. Residues highlighted by the red box are the conserved SAM binding motif (motif I). The small pink triangles indicate the residues in contact with SAM and substrates (JHA or FA). Secondary structure elements of AmJHAMT are showed as labeled blue cylinders (α helices) and brown arrows (β strands).

The full length cDNA sequence of *AmJHAMT* is 1253bp long, including a 285bp 5’ UTR and a 131bp 3’ UTR that contains a polyA signal (AATAAA) and a 31bp polyA tail. The *AmJHAMT* ORF encodes a protein of 278 amino acids with a predicted molecular weight of 33.0032 kDa and a theoretical isoelectric point of 5.86. No signal peptide cleavage sites were detected in the amino acid sequence, indicating that it is a non-secretory protein. Sequence alignment showed that AmJHAMT shared 36, 34, 32, 32, 35 and 32% amino acid identity with the reported JHAMTs from *Drosophila melanogaster* (GenBank accession number: NP_609793), *Bombyx mori* (NP_001036901), *Samia cynthia ricini* (ABE98256), *Aedes aegypti* (XP_001651876), *Schistocerca gregaria* (ADV17350), and *Tribolium* *castaneum* (EFA02917) (Figure 1), respectively. The motif I hh(D/E) hGxGxG is highly conserved in the SAM-MT superfamily, where h represents a hydrophobic residue [33]. Motif I was also found in AmJHAMT as CLDIGCGPG (Figure 1). In addition, active sites spatially bound with SAM and substrates (JHA or FA) have been identified in several JHAMTs [34]. Most of these active sites are also conserved in AmJHAMT (Figure 1). The core fold of SAM-MTs is composed of alternating α helices and β strands [35]. This typical fold was also detected in the secondary structure of AmJHAMT, which incorporated alternating 9 α helices and 6 β strands (Figure 1).

The full length sequence of *AmJHAMT* was submitted to GenBank and received an accession number KC335148.

### Phylogenetic analysis of JHAMT homologs

**Figure 2:**
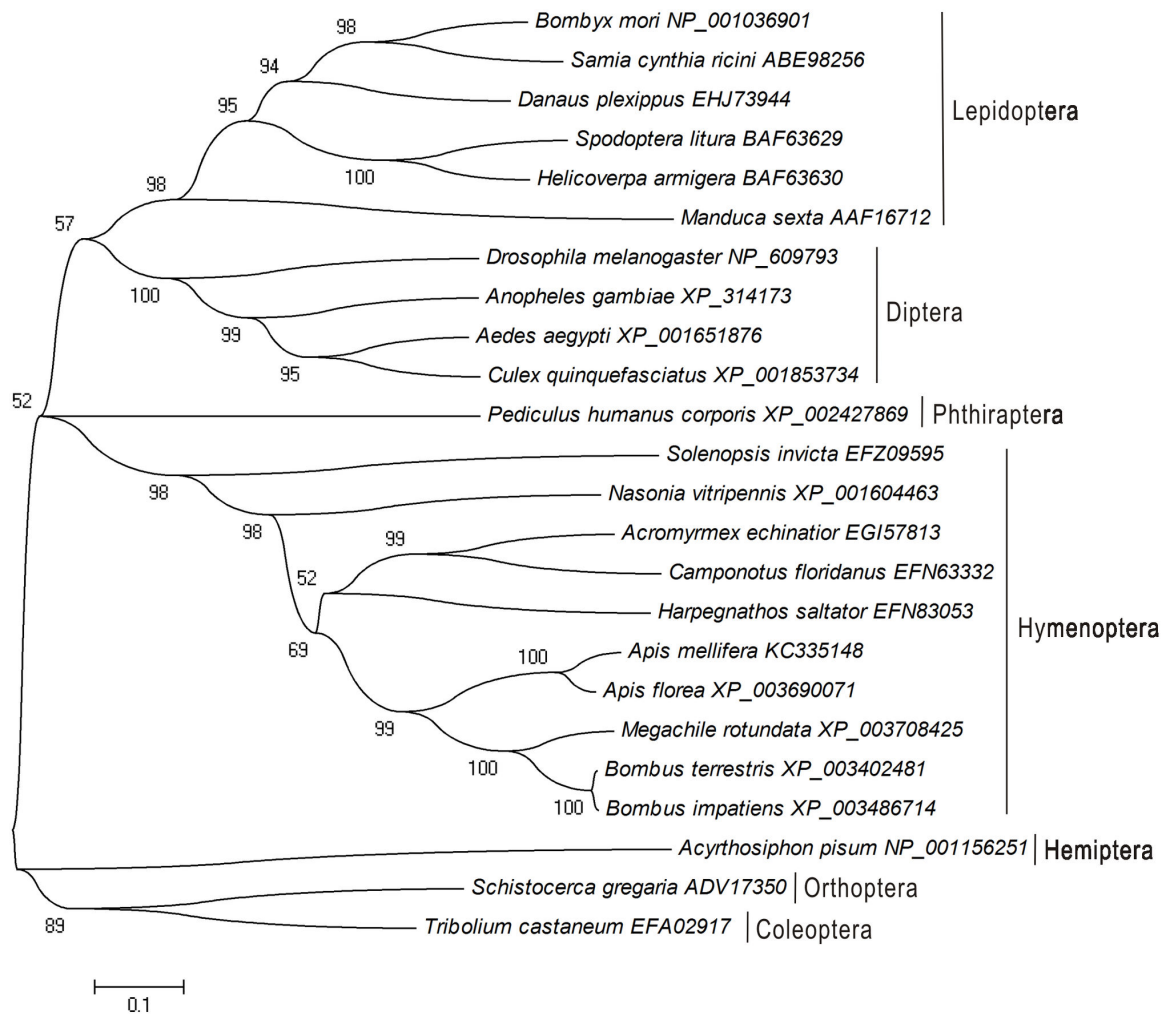
Phylogenetic analysis of JHAMT homologs from different insect species based on amino acid sequences. Sequences are labeled with the species Latin names plus GenBank accession numbers. Bootstrap values (1000 replicates) are displayed by the nodes. The genetic distance is drawn to scale.

Many JHAMT orthologs are found in insects. To investigate the phylogenetic relationship of these orthologs, we constructed a phylogram based on 24 JHAMT protein sequences from seven insect orders (Figure 2). Phylogenetic analysis indicated that AmJHAMT shared the highest similarity with four orthologs from species of Apoidea: *Apis* *florea* , *Bombus* *terrestris* , *Bombus* *impatiens* , and *Megachile* *rotundata* . Their pairwise similarities to *A. mellifera* were 89%, 64%, 64%, and 61%, respectively. The branch formed by these five orthologs was clustered with those from ants and parasitoid wasps, which, together, generate a major clade. The sampled taxa from Lepidoptera and Diptera formed clusters and are sister to one another. Two orthologs from Orthoptera and Coleoptera clustered to produce a third major clade and the rest of the major clades were formed by sampled taxa from Phthiraptera and Hemiptera.

### Verification of recombinant AmJHAMT

**Figure 3:**
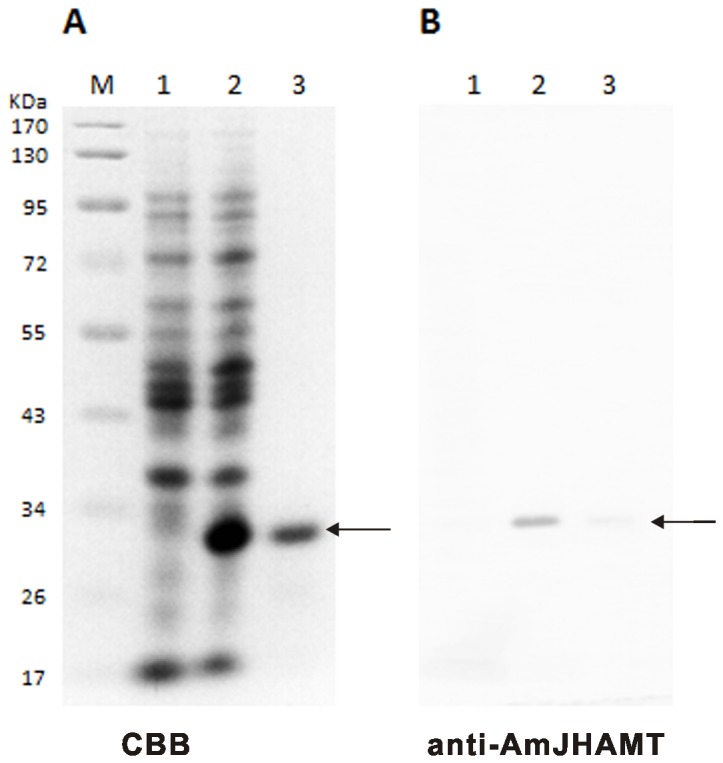
Preparation of the recombinant AmJHAMT protein expressed in *E. coli*. (A) Coomassie brilliant blue (CBB) staining of recombinant AmJHAMT. Samples were: lane 1 (negative control), crude supernatant from BL21(DE3)/pET28a (empty vector); lane 2, crude supernatant from BL21(DE3)/pET28a(+)/AmJHAMT; and lane 3, recombinant AmJHAMT (marked by arrow) purified from the product shown in lane 2. (B) Western blotting using polyclonal anti-AmJHAMT antibody. Samples are the same with A. Arrow indicates the recombinant AmJHAMT. M shows the protein ladder and the molecular weight of its single band is listed on the left.

We used the *AmJHAMT* sequence and an *E. coli* expression system to generate a recombinant AmJHAMT. The recombinant protein was purified and the anti-AmJHAMT polyclonal antibodies were subsequently prepared using the purified protein as an antigen. We analyzed the expression of recombinant AmJHAMT by SDS-PAGE and western blotting (Figure 3). The anti-AmJHAMT polyclonal antibody (diluted 1:32000) was used and also tested in the immunoblotting experiment. The SDS-PAGE and western blot showed that the recombinant AmJHAMT was significantly expressed and had a molecular weight of approximately 33 kDa. Moreover, western blotting indicated that the anti-AmJHAMT antibody was of high titer and specificity.

**Figure 4:**
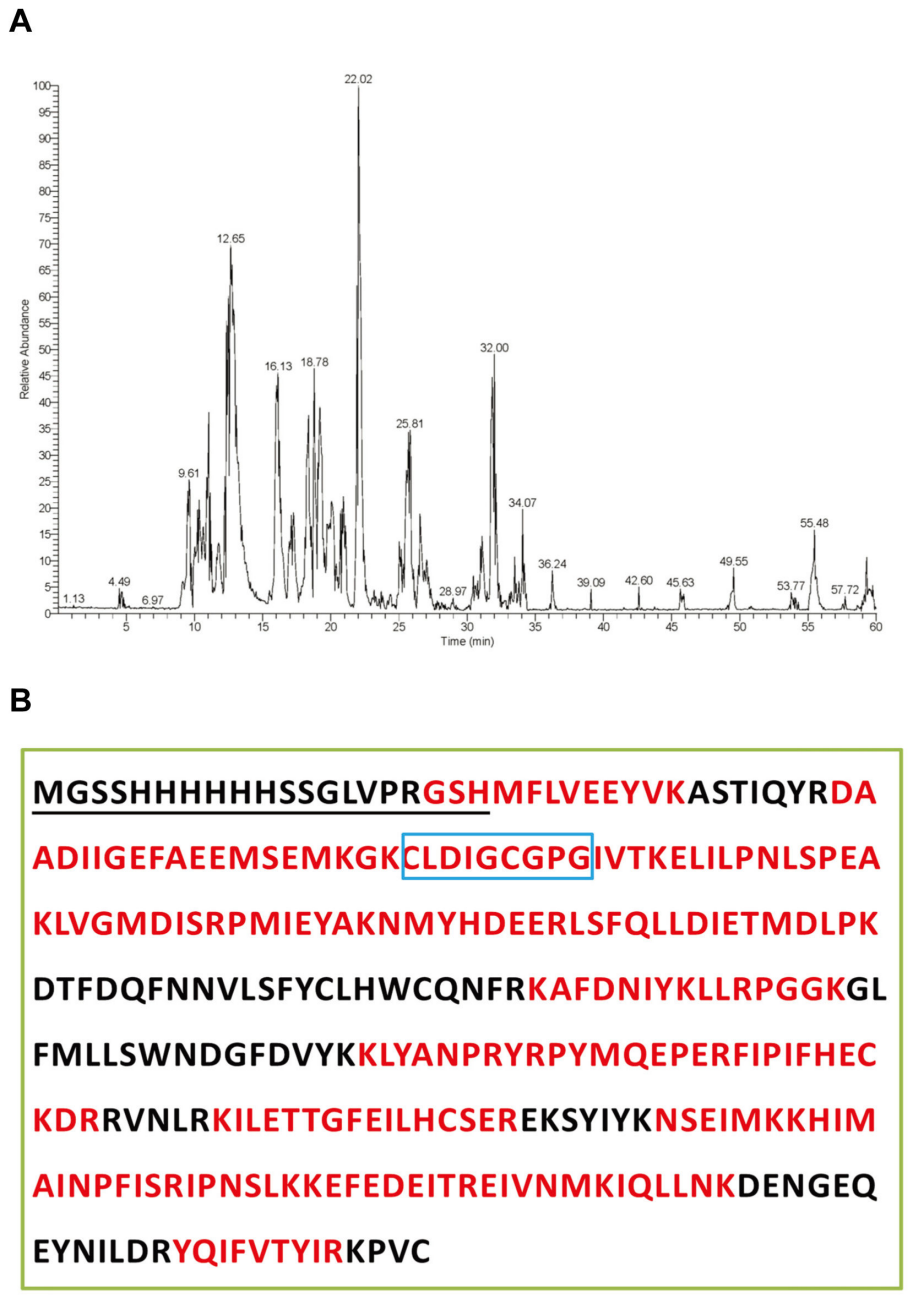
Verification of the AmJHAMT amino acid sequence by LC-MS/MS. (A) Total ion chromatograms (TIC). Numbers on the top of peaks indicate the retention time of a single peptide. (B) Sequence coverage of recombinant AmJHAMT by identified peptides. The red characters indicate amino acid residues covered by LC-MS/MS peptides. Characters underlined show the additional sequence from expression vector. Characters marked with blue box indicate the motif I.

To verify the sequence of recombinant AmJHAMT further, we conducted LC-MS/MS analysis on the purified protein. About 70% of the deduced recombinant AmJHAMT sequence was covered with the peptides detected by LC-MS/MS, while we obtained less than 7% sequence coverage with other proteins of *Apis mellifera* (Figure 4). Motif I mentioned above was also detected.

### Expression profiles of *AmJHAMT* gene and protein in queens and workers

**Figure 5:**
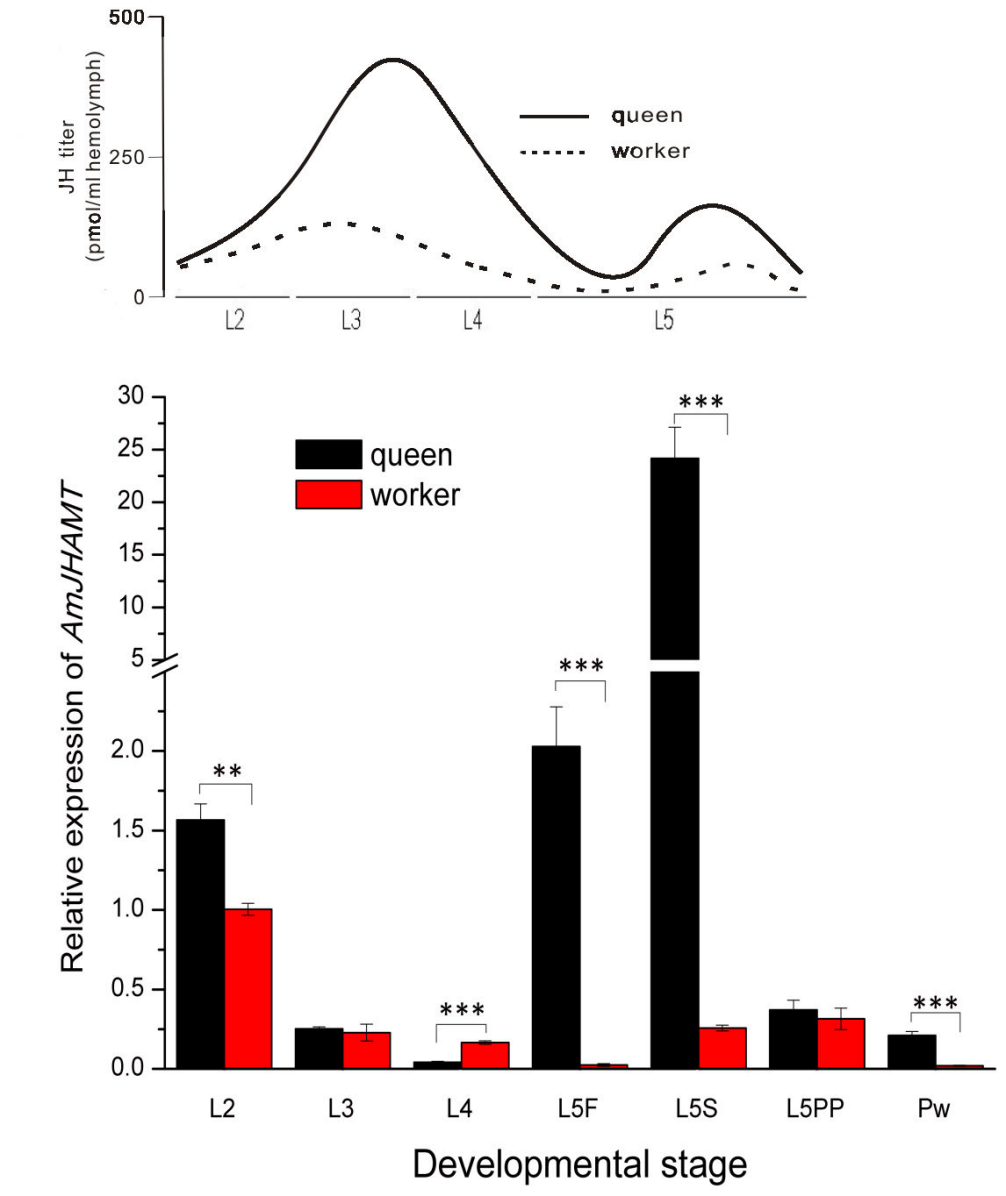
Quantitative real-time PCR analysis of *AmJHAMT* expression during honey bee caste development. The study covered early larval stage to initial pupal stage, including the second (L2), third (L3), fourth (L4), fifth (L5) instar larvae and white-eyed pupae (Pw). The L5 was subdivided into three stages known as the feeding stage (L5F), spinning stage (L5S), and prepupal stage (L5PP). Values are expressed as mean ± SEM (n = 3). Statistically significant differences are indicated by asterisks (independent-sample *t*-test, ** *P*< 0.01, *** *P* < 0.001). JH titers displayed above are based on data from Hartfelder and Engels [2].

The mRNA expression profiles of *AmJHAMT* in queen and worker castes were examined by qPCR analysis. Developmental stages from young larvae to early pupae were sampled except L1, as L1 larvae had to be transferred to rear queens. There was more abundant *AmJHAMT* mRNA in queens than that in workers in almost all developmental stages. Significantly higher levels were found in the L2 stage (*t*= 5.62, df = 4, *P*=0.006), L5F stage (*t*=7.98, df = 4, *P*<0.001), L5S stage (*t*=8.05, df = 4, *P*<0.001), and Pw stage (*t*=7.93, df = 4, *P*<0.001). Lower levels of *AmJHAMT* mRNA in queens were found only in the L4 stage (*t*=-9.56, df = 4, *P*<0.001). After the queen larvae developed into L5 stage, the expression of *AmJHAMT* dramatically increased and reached its highest level, which was about 100 times higher than that in workers (Figure 5). The expression of *AmJHAMT* decreased to very low levels in both queens and workers from the end of L5 to Pw (Figure 5).

**Figure 6:**
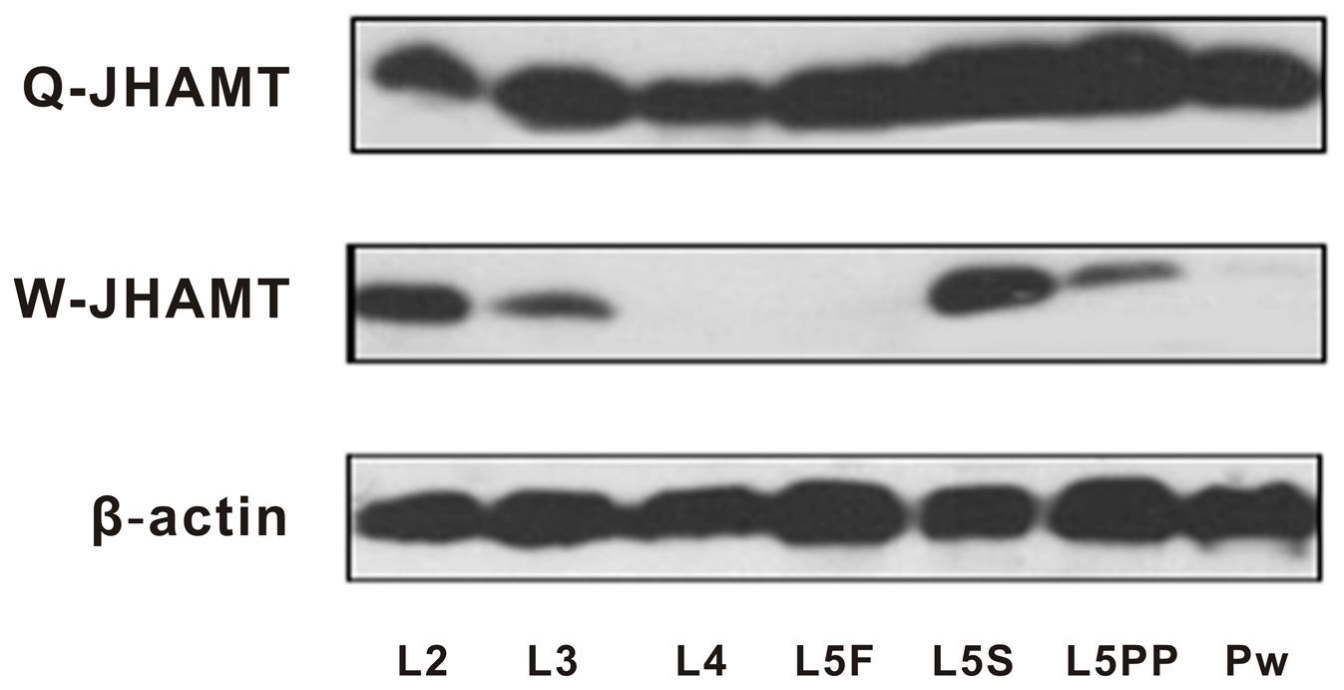
Immunoblotting analysis of AmJHAMT protein level during honey bee caste development. Q- and W-JHAMT mean the AmJHAMT protein expressed in queen larvae and in worker larvae, respectively. β-actin was set as an internal reference. The abbreviations of the developmental stages are as follows: L2, the second instar; L3, the third instar; L4, the fourth instar; L5F, the feeding stage of the fifth instar; L5S, the spinning stage of the fifth instar; L5PP, the prepupal stage of the fifth instar; Pw, the white-eyed pupa.

Queens also had more abundant AmJHAMT protein than workers in almost all developmental stages (Figure 6). In the L4 and L5F stages especially, AmJHAMT was barely detected in worker larvae, but noticeable levels of this protein were present in queen larvae (Figure 6). This period is thought to be JH-sensitive and a physiologically critical temporal window [2]. Keeping AmJHAMT protein available at this period might be important for the induction of queen development. For queen and worker bees, the protein levels declined from the end of L5 to Pw. But unlike workers, the queens sustained a relatively high level of AmJHAMT when they underwent metamorphosis (Figure 6).

A summary of *AmJHAMT* gene and protein expression profiles in queens and workers reveals that two sequential peaks were present in all profiles (Figure 5 and Figure 6) and a similar pattern was seen in the JH titer profiles [2] (Figure 5).

## Discussion

Many biological methylation reactions are catalyzed by members of the SAM-MT superfamily, which use SAM as the methyl donor to methylate substrates [35]. Based on the substrate specificity, SAM-MTs are classified as DNA, RNA, protein, lipid, and small-molecule methyltransferases (MTs). Although SAM-MTs usually share a very low sequence similarity, several conserved motifs have been identified. The presence of motif I (CLDIGCGPG) indicates that AmJHAMT belongs to the SAM-MT superfamily, similar to previously-described JHAMT orthologs in the silkworm, mosquito, and desert locust [10,12,13].

The structures of SAM-MTs are highly similar across insects. All reported SAM-MTs have a common core fold, composed of alternating β strands (β1-β7) with α helices (αZ and αA-αE). The topology of the core fold shows that the SAM-binding region is usually located close to the N-terminal part of a polypeptide, and the substrate-binding region is always localized near the C-terminal [35]. However, the individual substrate-binding regions vary greatly due to the need to bind different substrates [35]. A typical SAM-MT fold was found by analyzing the secondary structure of AmJHAMT, which incorporates a 6-stranded β sheet with 9 α helices. The expected topology of the fold of AmJHAMT was close to that known from the lipid SAM-MT fold. This suggests that lipids are the source of the substrates of AmJHAMT. In fact, known JHAMTs all use juvenoid acids (JHA or FA) as substrates and cannot catalyze other fatty acids, such as palmitic acid and lauric acid, suggesting that they have a relatively high substrate specificity [10,13-15].

Additionally, the secondary structures shared by AmJHAMT and other JHAMT orthologs like AaJHAMT (*A. aegypti*), BmJHAMT (*B. mori*), and DmJHAMT (*D. melanogaster*) are highly consistent. The computational tertiary structure of JHAMTs visually demonstrates the interactions among the enzymes, SAM and substrates. Several active sites have been identified in JHAMTs [34]. In AaJHAMT, Asp-69 and -41 bind SAM by hydrogen bonds, and Val-70 and Ile-95 form a hydrophobic pocket where the adenine ring of SAM is located. Gln-14 and Trp-120 bind the carboxyl group of FA or JHA and place them in a suitable conformation for catalysis. Another hydrophobic pocket, formed by Ile-151, Ile-154, Tyr 155, Leu-158, Val-221, and Val-224, is used to load the carbon chains of juvenoid acids. The last two residues, Ser-176 and Tyr-178, bind the epoxide group of (10R)-JHA [34]. Most of the active sites of AaJHAMT are also conserved in AmJHAMT; however, Ile-151 and Ser-176 are substituted by Gly-146 and Pro-171, respectively (Figure 1). Gly is smaller and less hydrophobic than Ile and Pro has a weaker ability to form hydrogen bonds than Ser. These properties seem detrimental to the common functions of JHAMTs. However, because the majority of the active sites do not change, the overall environment remains hydrophobic and stable enough for the loading of substrates and the enzyme is still able to catalyze JHA/FA.

In *Apis mellifera*, fertile queens and sterile workers are alternative forms of adult females. This dimorphism does not depend on genetic differences but on the differential feeding of royal jelly [36]. Nutritional differences at the larval stage trigger endocrine responses and JH is the primary endogenous signal inducing queen development [2]. Topical application of JH and its analogs on the fourth and early fifth instar worker larvae induces development as queens or at least expression of some queen-like characteristics [37-39]. Studies on JH biosynthesis and titer measurements also support the queen-induction property of JH [40,41]. Titers of JH in queen larvae are much higher than those in worker larvae throughout most of their larval development [41] and expression levels of *AmJHAMT* in queen larvae are consistently and significantly higher than those in worker larvae throughout most developmental stages (Figure 5).

There are two peaks of JH titer in queen larvae, one located near the end of the third instar, and the other at the middle of the prepupal stage. The former one is much higher than the latter [40,41]. Higher levels of JH titer in the fifth instar queen larvae prevent their ovaries from apoptosis, yielding fully-developed ovaries. Meanwhile, the ovaries of worker larvae undergo programmed cell death [42]. In the expression profile of *AmJHAMT* in queen larvae, two peaks also emerge, but they differ from those of JH titer in two ways (Figure 5). First, these two peaks are elevated slightly earlier than those in JH titers. Considering the time between gene expression and significant JH biosynthesis and release, we would expect to see gene expression differences prior to actual JH titer changes. Second, in the gene expression profile, the former peak is much lower than the latter, in contrast to the two peaks in JH titers. Although higher *AmJHAMT* transcription could enhance JH biosynthesis, JH metabolism might also significantly increase at the same time. In fact, the juvenile hormone esterase gene (*jhe*) is differentially expressed in the developmental stages of worker larvae and can effectively reduce the JH titer to some extent [43]. Regardless of these differences, there is a similar pattern between the expression profile of *AmJHAMT* and JH biosynthetic activity during queen and worker larvae development (Figure 5). Considering the critical role of JH in queen induction, AmJHAMT may serve as an important regulatory element controlling caste differentiation.

In general, a gene’s mRNA level cannot determine its final protein expression. Compared with mRNA levels, protein abundance may more directly reflect a gene’s functional response to a distinct biological status. Therefore, it was necessary to further profile AmJHAMT protein expression during the same developmental stages of both queen and worker bees. We performed an immunoblotting analysis to determine the protein expression profiles. The results confirm two things: First, the *AmJHAMT* gene and protein were both differentially expressed during queen and worker larvae development, which lends further support for a close relationship between *AmJHAMT* and caste differentiation. Second, there was a similar pattern among gene expression, protein expression, and JH biosynthesis although the profiles did not match as closely as expected.

Insect larval-pupal metamorphosis is a hormone-controlled process. When a larva attains a proper size in the final instar, JH titers in the hemolymph decrease rapidly. This results from the cessation of JH biosynthesis and a rise in JH metabolism and ecdysteroid titers, triggering metamorphosis [3]. Since JH biosynthesis ends during metamorphosis, JH synthetic enzymes are down-regulated. Significant suppressions of *JHAMT* gene transcriptions were reported during the larval-pupal metamorphosis in silkworms [10], fruit flies [15], red flour beetles [14], mosquitos [13], and desert locusts [12]. In this experiment, a similarly low expression level of *AmJHAMT* was found in both queen and worker bees during metamorphosis (Figure 5). The protein level during this process declined rapidly to a very low level in worker bees, but not in queen bees. The reason why queens sustain a relatively high level of AmJHAMT protein during metamorphosis should be studied further.

In summary, our results find that the *AmJHAMT* gene cloned from honey bee larvae encodes a juvenile hormone acid methyltransferase that catalyzes the final methylation of JH biosynthesis. Phylogenetic analysis shows that AmJHAMT is clustered with all other Hymenopteran JHAMT orthologs to form an independent major clade. Significant differences between the temporal *AmJHAMT* mRNA and protein expression profiles in queen and worker larvae suggest that proper developmental regulation of this gene is crucial for female caste differentiation in *Apis mellifera*. Additionally, remarkable down-regulation of *AmJHAMT* from the end of L5 to Pw in both queens and workers indicates that suppression of this gene might play an important role in the metamorphosis of honey bees. More studies are needed to further explore these functions of *AmJHAMT* in honey bee physiology.

## Supporting Information

Figure S1 Tests on the PCR amplification efficiency of primer pairs. Serial 8× dilutions of cDNA samples were used as templates to generate relative standard curves. The regression equation, determination coefficient (*r* ^2^) and PCR amplification efficiency (*E*) were calculated and presented. A) test on the primer pair for the target gene *AmJHAMT*; B) test on the primer pair for the reference gene *actin*.
(PDF)
