# Supplementary figures and images for: Molecular Cloning and Characterization of Juvenile Hormone Acid Methyltransferase in the Honey Bee, Apis mellifera, and Its Differential Expression during Caste Differentiation
Source: PLoS One. 2013 Jul 9;8(7):e68544. doi: 10.1371/journal.pone.0068544 (PMC3706623; doi:10.1371/journal.pone.0068544)

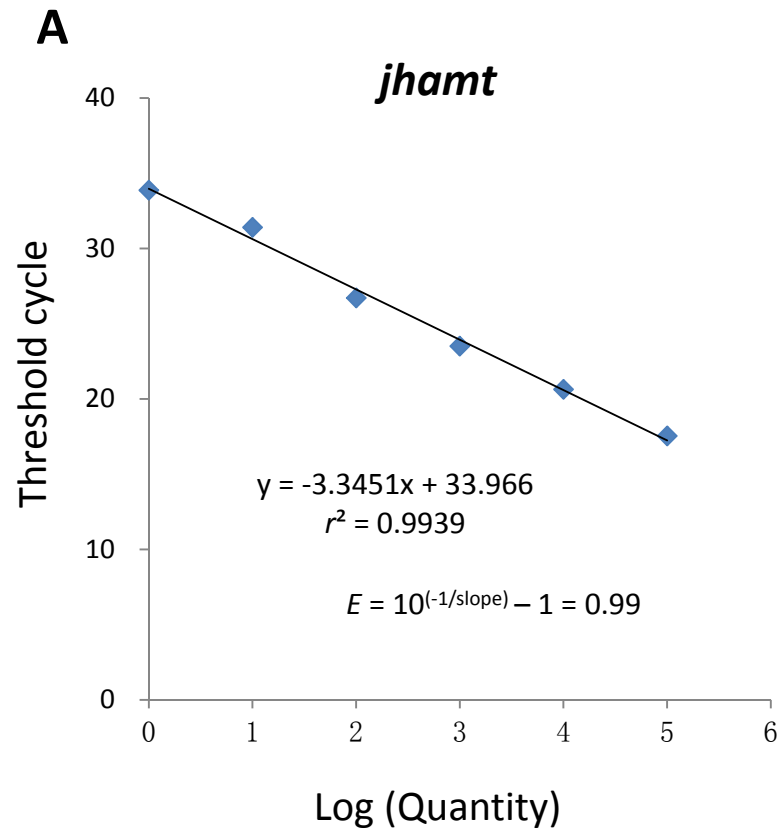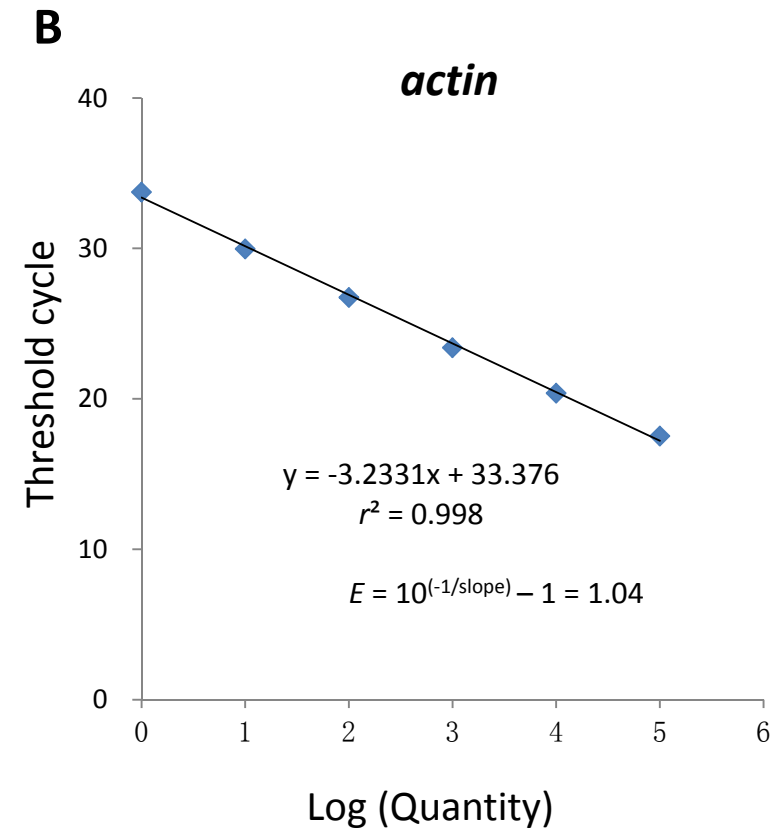

Supplement: Figure S1 — Serial 8× dilutions of cDNA samples were used as templates to generate relative standard curves. The regression equation, determination coefficient (r 2) and PCR amplification efficiency (E) were calculated and presented. A) test on the primer pair for the target gene AmJHAMT; B) test on the primer pair for the reference gene actin. (PDF) [file pone.0068544.s001.pdf]
